# Testicular Neuroendocrine Tumors: A Case Report and Literature Review

**DOI:** 10.7759/cureus.37370

**Published:** 2023-04-10

**Authors:** Abdulaziz A Albalawi, Abdalatiff K Bedaiwi, Mutlaq A Alotaibi, Khalid Bedaiwi

**Affiliations:** 1 Urology, Prince Sultan Military Medical City, Riyadh, SAU

**Keywords:** mediastinal lymph node metastasis, testicular neuroendocrine tumor, tnet, carcinoid tumor, neuroendocrine tumors, testicular cancer

## Abstract

Testicular neuroendocrine tumors (TNETs) are extremely rare. We report a case of a primary TNET and discuss the clinical and histological characteristics, treatment, and prognosis of this tumor. A 47-year-old man had a painless right testicular mass. All tumor markers were negative. The patient underwent a high inguinal radical orchidectomy. Histopathology revealed a well-differentiated neuroendocrine tumor. Radiological investigations showed multiple prominent axillary, supraclavicular, mediastinal, and hilar lymph nodes and no bowel or mesenteric lesions suggesting carcinoid. Once a TNET is diagnosed, it is necessary to rule out the secondary origin in the gastrointestinal tract and lungs. Radical orchiectomy is the treatment of choice for TNETs. Somatostatin analogs can be useful in patients with carcinoid syndrome, induce symptomatic improvement, and control disease progression. As this case highlights, physicians should consider TNETs in the differential diagnosis of testicular masses, as early diagnosis and treatment are crucial for good patient outcomes.

## Introduction

Neuroendocrine tumors (NETs) are a group of malignancies arising from neuroendocrine cells throughout the body, usually called carcinoid tumors [1]. The majority of NETs can present in the gastrointestinal tract (85%). Other sites include the lung, biliary tract, pancreas, ovaries, thymus, and rarely, the testes [2]. Testicular neuroendocrine tumors (TNETs) represent less than 1% of all testicular tumors [3]. We present a case of a primary TNET in Saudi Arabia, discuss the clinical and histological characteristics, diagnosis, treatment, and prognosis of this rare tumor, and present a brief literature review of similar cases.

## Case presentation

A 47-year-old man with a history of diabetes, hypertension, stage 3 chronic kidney disease, hypothyroidism, atrial fibrillation, and severe tricuspid regurgitation with a history of tricuspid valve replacement was admitted to the Prince Sultan Cardiac Centre with right-sided heart failure and type 2 respiratory failure. The patient was referred to the urology department with the complaint of a painless right testicular mass for eight months. A non-tender, firm right testicular mass was noted on physical examination, but no enlarged lymph nodes were palpated in the groin. His Eastern Cooperative Oncology Group Performance Status Scale score was 3.

**Figure 1:**
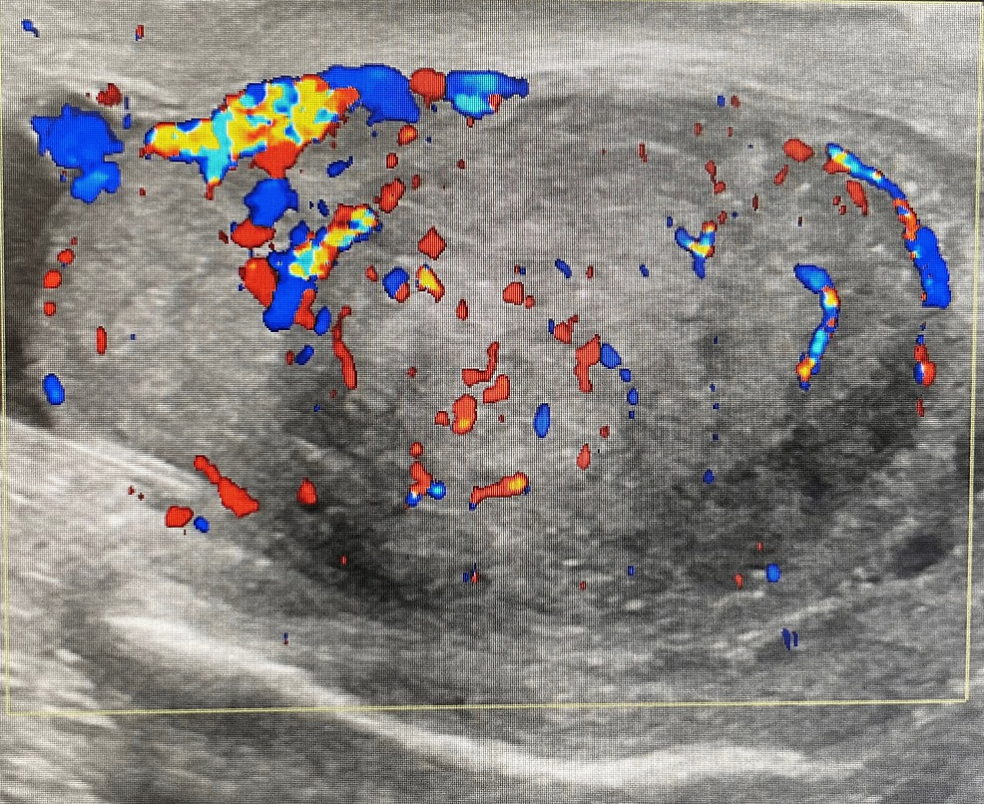
Scrotal ultrasound showing the right testis is enlarged, measuring 3.5 cm x 3.1 cm x 5 cm, demonstrating heterogeneous parenchymal echotexture occupied by a large vascular intratesticular heterogenous hypoechoic lesion measuring 3 cm x 3.6 cm x 3.4 cm. No calcification seen. The right epididymis appears to be swollen with normal vascularity.

His scrotal ultrasound revealed a large vascular intratesticular heterogeneous hypoechoic lesion on the right side measuring 3 cm x 3.6 cm x 3.4 cm without calcification (Figure 1).

All tumor markers were negative, with beta-human chorionic gonadotropin (β-HCG) < 1 mUI/mL, alpha-fetoprotein (AFP) 0.8 ng/mL, and lactate dehydrogenase (LDH) was 117 U/L. The patient subsequently underwent a high inguinal radical orchidectomy. Histopathological findings showed a well-differentiated NET with unifocal tumor focality and limited tumor extension to the testes. Lymphovascular invasion was not identified, and the surgical margin was negative for tumor tissue. Additionally, the vas deferens and epididymis were unremarkable. The tumor size was 3.5 cm in its greatest dimension.

**Figure 2:**
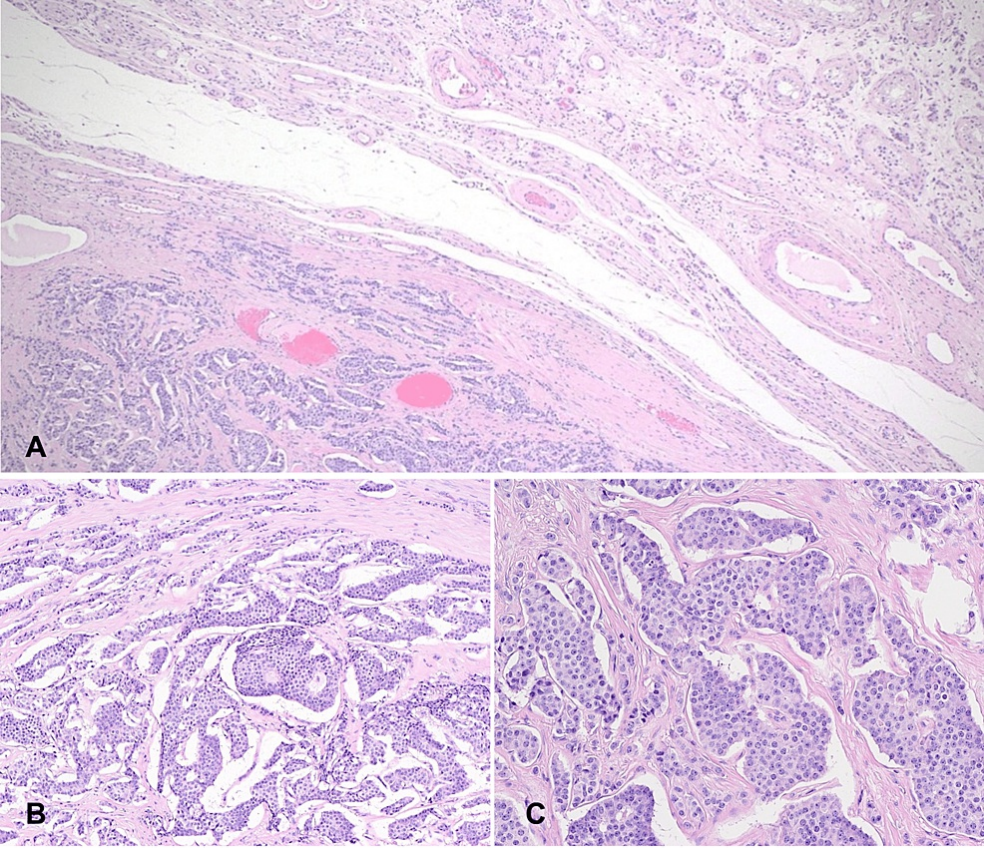
Histopathological examination of the specimen. (A) Low-power view showing well-defined neoplastic tumor with adjacent normal testicular tissue. (B) Intermediate-power view showing neoplastic cells arranged in nests, cribriform, microglandular patterns. (C) High-power view of tumor cells showing round monotonous cells with coarse, salt and pepper nuclear chromatin - no atypia, necrosis, or mitosis.

**Figure 3:**
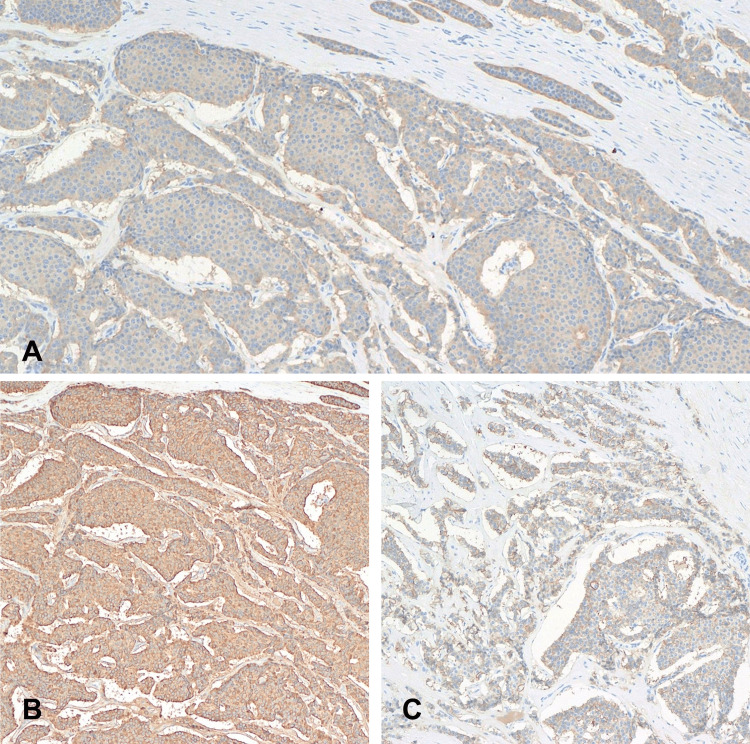
Immunohistochemical examination of the specimen shows positive staining for synaptophysin (A), chromogranin A (B), and CD56 (C).

The histological sections demonstrated tumor cells arranged in nests in trabecular, insular, and microcystic patterns (Figure 2). The tumor cells were monotonous regular cells with round nuclei with salt and pepper chromatin and moderate eosinophilic granular cytoplasm. No mitotic activity or necrosis was seen. A large panel of immunohistochemical stains was done, which showed positivity for synaptophysin, chromogranin, CD56, AFP (focal), OCT4 (focal), and panCK, and negative for inhibin, calretinin, D2-40, CD117, CD30, glypican 3, prostate-specific antigen, and Ki67 index < 1% (Figure 3). The overall histological and immunophenotypic features suggested a well-differentiated NET (TNET prepubertal type).

Further imaging studies included a chest computed tomography (CT) scan that showed multiple prominent axillary, supraclavicular, mediastinal, and hilar lymph nodes. The abdominal and pelvic CT scan showed no bowel or mesenteric lesions, suggesting carcinoid. A gallium-68 Dotatate whole-body positron emission tomography/CT scan showed multiple bilateral prominent supraclavicular, axillary, bilateral hilar, right paratracheal, paraaortic, and subcarinal lymph nodes showing radiotracer activity most evident in the right hilar region. The medical oncology team evaluated the patient, but he was not fit to receive chemotherapy due to the comorbidities mentioned above. Given that he lived in a remote area, he was unavailable for follow-up with our care team. The patient decided to follow up at his hometown hospital.

## Discussion

NETs were first described in 1867 by Langhans [1] and the primary TNET was first reported by Simon et al. in 1954 [2]. Primary TNETs account for only 0.23% of all testicular tumors [3]. According to the 2022 World Health Organization testicular tumor classification system, TNETs are germ cell tumors unrelated to germ cell neoplasia. The term “carcinoid” has been replaced with “neuroendocrine tumor,” and those in the testis are now classified as “prepubertal type testicular neuroendocrine tumors” [4].

Carcinoid tumors occur in the testes as (1) primary pure testicular carcinoid; (2) primary with epidermoid or dermoid cysts; (3) primary associated with teratoma; and (4) metastatic carcinoid to the testis [5]. Amine et al. performed a meta-analysis and reviewed 132 cases with TNETs from 1930 to early 2015. The median age at diagnosis was 39 years (range 10 to 83 years). A testicular mass or swelling was the most common presenting symptom in 38.46% of cases. Serum tumor markers (β-HCG, AFP, and LDH) were within reference ranges in all patients except one case. About 76% of TNETs were primarily pure type. All tumors tested positive for chromogranin and synaptophysin. Eight cases (6.06%) had metastatic disease at the time of diagnosis. Radical orchiectomy was the primary treatment in 127 patients (96.21%). The five-year overall survival rate was 78.70% [6].

Only one case of primary TNET associated with mediastinal lymph node metastasis was found in the literature. It was reported by Hosking in 1981 for a 27-year-old man diagnosed with primary TNET and treated with radical orchidectomy. Seventeen years after the initial diagnosis, he presented with liver and cervical lymph node metastasis. Mediastinal lymph node metastasis was only discovered after autopsy [7].

The treatment of choice for TNET is radical inguinal orchiectomy with close follow-up [8]. However, if associated with teratoma, the treatment is similar to testicular teratoma. The indications for retroperitoneal lymph node dissection in pure primary TNET remain unclear [9].

Adjuvant treatments for TNET include chemotherapy, radiation therapy, somatostatin analogs, and α-interferon. The current protocol for adjuvant chemotherapy for metastatic NETs includes 5-fluorouracil, streptozocin, doxorubicin, or cyclophosphamide. However, this protocol generates only short responses in <10% of patients. Radiation therapy has little effect on metastatic NETs [6]. Octreotide, a somatostatin analog, showed symptomatic improvement in 80% of patients diagnosed with metastatic disease and can stop tumor growth, according to a meta-analysis that studied the effect of somatostatin analogs on NETs [10].

## Conclusions

TNETs are extremely rare but overall have a good prognosis. Once a TNET is diagnosed, it is necessary to rule out the secondary origin in the gastrointestinal tract and lungs. The treatment of choice is radical inguinal orchiectomy with close follow-up, while the indications for retroperitoneal lymph node dissection in pure primary TNET remain unclear. Adjuvant treatments include chemotherapy, radiation therapy, somatostatin analogs, and α-interferon. As this case highlights, physicians should consider TNETs in the differential diagnosis of testicular masses, as early diagnosis and treatment are crucial for good patient outcomes.
